# Chemical Modification and Performance Evaluation of *Eucommia ulmoides* Gum as a Natural and Sustainable Energy Resource and Its Application in Road Engineering

**DOI:** 10.3390/polym18091030

**Published:** 2026-04-24

**Authors:** Shichao Cui, Naisheng Guo, Jun Zhang, Guangshuai Wu, Hongbin Zhu, Yiqiu Tan

**Affiliations:** 1 College of Transportation Engineering, Dalian Maritime University, Dalian 116026, China; cuishichao1000@126.com (S.C.); zhangjun123@dlmu.edu.cn (J.Z.); wgs123456@dlmu.edu.cn (G.W.); 2 School of Transportation Science and Engineering, Harbin Institute of Technology, Harbin 150090, China; tanyiqiu@hit.edu.cn

**Keywords:** EUG, chemical modification of EUG, renewable energy, modified asphalt, rheological properties, AFM

## Abstract

*Eucommia ulmoides* gum (EUG), a sustainable plant-derived natural polymer, was functionalized via three distinct routes, including vulcanization, epoxidation, and hydroxylation to yield vulcanized (VEUG), epoxidized (EEUG), and hydroxylated EUG (HEUG), respectively. We systematically characterized the effects of modification route and degree on the chemical structure, crystallization behavior, thermal stability, hydrophilicity, and mechanical properties of functionalized EUG and further evaluated the high/low-temperature performance, microstructure, and mechanical properties of the corresponding modified asphalt binders (VEMA, EEMA, HEMA) as a function of modifier type and loading. For VEUG, C-S cross-linking networks formed during vulcanization suppress EUG crystallization, enabling a rigid-plastic to elastic transition, while high-temperature cleavage of C-S bonds reduces its initial thermal stability. For EEUG, epoxidation breaks C=C double bonds and introduces epoxy groups to strengthen intermolecular interactions; subsequent ring-opening grafting of hydroxyl groups onto EEUG yields HEUG, which forms additional cross-links via dynamic hydrogen bonds. Increasing modification degree for both EEUG and HEUG reduces their number- and weight-average molecular weights with narrower distribution, diminishes crystallinity, enhances thermal stability and hydrophilicity, and drives a rigid-plastic to elastic transition, characterized by decreased strength (0.65 MPa < σ_HEUG_ < σ_EEUG_ < 10.18 MPa) and markedly improved ductility (143.6% < ε_EEUG_ < 262.0%, 679.9% < ε_HEUG_ < 1360.3%). In asphalt binders, VEUG’s cross-linked network endows VEMA with refined more abundant bee-like microstructures, drastically boosting high- and low-temperature performance: relative to pristine EUG-modified asphalt (EUGMA), VEMA’s DMT modulus decreases by 94%, and adhesion increases by 87%. EEMA forms covalent bonds with polar asphalt components via epoxy groups, while HEMA constructs a hydrogen-bonded cross-linked network; both effectively inhibit asphaltene aggregation. With increasing modifier loading, EEMA and HEMA exhibit increased modulus, reduced adhesion, and gradually improved high- and low-temperature performance, except for the non-significant high-temperature enhancement of HEMA at higher loadings.

## 1. Introduction

Asphalt pavement is renowned for its excellent flatness, low noise, and superior driving comfort and thus has become one of the mainstream pavement types worldwide. However, asphalt pavement nowadays is subjected to the characteristics of heavy vehicle axle load, a short load application time, and a high traffic volume, which often leads to distress such as rutting, potholes, and cracking [1,2]. To address these problems, modified asphalt pavement has been widely adopted. Currently, commonly used asphalt modifiers include styrene–butadiene–styrene block copolymer, waste rubber powder (CR), and polyphosphoric acid, etc. [3,4,5]. Nevertheless, most of these modifiers are by-products of the petroleum industry; their prices fluctuate significantly with the crude oil market conditions, and they are classified as non-renewable resources. Therefore, it is particularly important to explore a green, sustainable, and renewable asphalt modifier.

*Eucommia ulmoides* gum (EUG), also known as gutta-percha or balata, is a natural, green and renewable polymer, whose main component is trans–1,4–polyisoprene, a structural isomer of natural rubber. The presence of trans C=C double bonds endows EUG with excellent chain flexibility, which makes it prone to crystallization at room temperature and thus exhibits plastic properties; this characteristic has severely restricted its practical applications [6]. To overcome the limitation of EUG’s high crystallinity, researchers have developed various modification methods, among which chemical modification is the most commonly adopted one. Specifically, approaches such as epoxidation [7], hydroxyl grafting [8], sulfonation [9], or the introduction of alderene groups to form intermolecular hydrogen bonds [10] can reduce the crystallinity, precisely regulate the transition of the material from plasticity and elasticity to viscosity, and endow it with properties including self-healing, reinforcement and toughening, or high elasticity and toughness. In addition, ring-opening cross-linking can impart shape memory function to EUG [11]. Secondly, vulcanization cross-linking is another key regulation method. The cross-linked network can inhibit crystallization [12] and significantly affect the dynamic mechanical properties of EUG, leading to specific regular correlations between the damping behavior in the glass transition region and melting region with the cross-linking degree and crystallinity, respectively [13]. In addition, physical blending can also effectively realize functional integration and performance optimization: blending EUG with low-density polyethylene [14], natural rubber [15,16], nitrile butadiene rubber [16], or epoxy resin [17] can construct a bicontinuous structure, thereby improving the damping performance, wear resistance and damage resistance, as well as achieving multi-temperature shape memory behavior. The application of foaming composite technology has further developed materials with both lightweight characteristics and excellent shape memory function [18]. These studies have revealed the intrinsic relationship between the molecular structure and properties of EUG through various chemical and physical modification methods, laying a solid foundation for its transformation from a plastic material to a multifunctional elastomer in practical applications.

In view of the highly tunable molecular structure and properties of EUG, researchers have actively explored its application in road engineering, with the focus on using it as an asphalt modifier to improve the service performance of pavement. Studies have shown that EUG and its modified derivatives can significantly enhance the service performance of asphalt. Li et al. [19,20] found that vulcanized EUG modified asphalt exhibited improved wear resistance, crack resistance, thermal stability, and flexibility, yet with reduced temperature sensitivity. Wang et al. [21] confirmed from a microscopic perspective that vulcanized EUG modified asphalt possessed favorable anti-aging performance. In terms of process optimization, Cui et al. [22,23,24,25] systematically investigated the preparation process and determined the optimal shearing and curing time, pointing out that the internally formed cross-linked network was the key mechanism endowing asphalt with excellent high-temperature deformation resistance, elastic recovery, and storage stability. Yan et al. [26,27,28] combined molecular dynamics simulation with rheological experiments, theoretically verifying the good compatibility among EUG, vulcanized EUG, and asphalt, as well as confirming that both types of modified asphalt exhibited outstanding high-temperature stability and deformation resistance. To further improve the performance of traditional waste CR-modified asphalt, researchers have also modified EUG and CR chemically to enhance their compatibility with asphalt. Deng et al. [29] adopted maleic anhydride-grafted EUG, which formed a three-dimensional network in CR-modified asphalt, significantly improving its elasticity, viscosity, rutting resistance, and storage stability. Shi et al. [30] achieved a compact composite of EUG and CR via high-temperature shear blending, thus enabling better chemical bonding with asphalt. These studies have systematically demonstrated the feasibility and application potential of EUG as a high-performance asphalt modifier, covering aspects ranging from performance verification and mechanism elucidation to process optimization.

**Figure 1:**
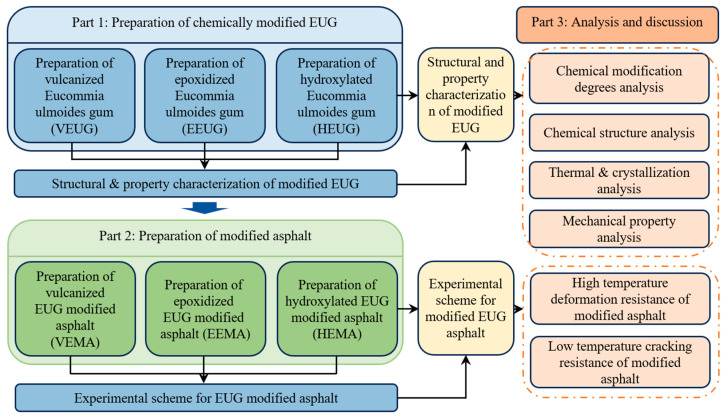
Technical flowchart.

Due to its tendency to crystallize at ambient temperatures, EUG often leads to poor low-temperature performance when incorporated into asphalt. Consequently, current research predominantly focuses on traditional modification methods, such as vulcanization or functional group grafting, to balance the high- and low-temperature properties of EUG-modified asphalt. However, there is limited research on modifying EUG at the molecular scale through chemical means to enhance its compatibility with asphalt. Furthermore, the modification mechanisms of EUG on asphalt vary significantly across different modification methods. In this study, three common chemical modification methods were selected based on the characteristics of EUG. By controlling the reaction conditions, vulcanized, epoxidized, and hydroxylated EUG samples with varying degrees of modification were synthesized. Their chemical structures, crystallization behavior, thermal stability, hydrophilicity, and mechanical properties were systematically characterized. On this basis, the various modified EUG samples were applied to asphalt modification to investigate the effects of modifier dosage on the high- and low-temperature pavement performance of the resulting asphalt binders. Their suitability under different climatic conditions was also evaluated. Finally, the underlying modification mechanisms were elucidated through a combination of micromorphological observation and mechanical property analysis. The technical flowchart is shown in Figure 1.

## 2. Raw Materials, Preparation, and Experimental Methods of Chemically Modified EUG and Its Modified Asphalt

### 2.1. Raw Materials

The experimental chemicals used for chemically modified EUG and their technical specifications are shown in Table 1.

The matrix asphalt (MA, PG64-28) used in this study is Grade 90 Type A petroleum asphalt, and its technical specifications are shown in Table 2.

### 2.2. Preparation of Chemically Modified EUG

#### 2.2.1. Preparation of Vulcanized *Eucommia ulmoides* Gum (VEUG)

**Figure 2:**
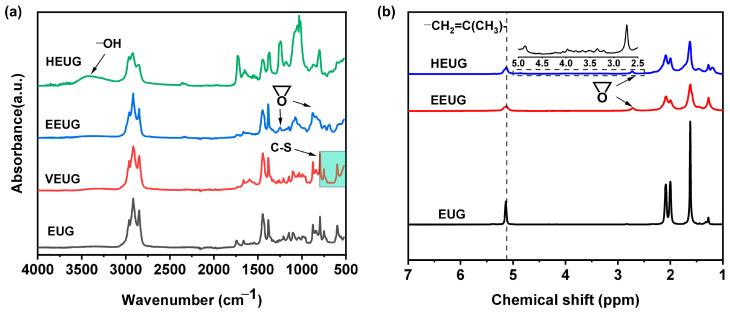
FTIR spectra (**a**) and ^1^H NMR spectra (**b**) of various modified EUG.

Natural EUG with a purity higher than 96% was selected as the raw material. The EUG was masticated on an open mill, where the mastication temperature was controlled within the range of 70~110 °C for approximately 20 min. Subsequently, ZnO, SA, CBS, IPPD, and sulfur were added sequentially in accordance with the formulation shown in Table 3. The mixing process was conducted by first thinning the rubber compound and then thickening it, with triangular wrapping repeated no less than 5 times to ensure homogeneous mixing. After sufficient mixing, the rubber sheet was prepared and allowed to stand for 12 h. An appropriate amount of the mixed rubber was taken and vulcanized in a mold using a 25-ton plate vulcanizing machine at a temperature of 150 °C and a pressure of 15 MPa to obtain VEUG. The VEUG samples prepared with different sulfur dosages were denoted as VEUG–*a*, where *a* represents the dosage of sulfur. The FTIR spectroscopy results of VEUG are presented in Figure 2a. It should be noted that VEUG is insoluble in any organic solvent due to the presence of cross-linked networks; thus, ^1^H NMR spectroscopy analysis cannot be performed. It can be observed that a characteristic absorption peak attributed to C–S bonds appears in the wavenumber range of 500~800 cm^−1^, which confirms the successful occurrence of the vulcanization cross-linking reaction.

#### 2.2.2. Preparation of Epoxidized *Eucommia ulmoides* Gum (EEUG)

EUG was added to the xylene solvent, followed by mechanical stirring to obtain an EUG solution. At 40 °C, a mixed solution of hydrogen peroxide (H_2_O_2_) and formic acid (HCOOH) was added to the EUG solution. The epoxy degree *E* (mol%) was controlled by adjusting the molar ratio of H_2_O_2_ to the C=C double bonds in EUG, where the molar ratio of [HCOOH]:[H_2_O_2_] =1:1. The reaction was allowed to proceed continuously for 5 h. Subsequently, the product was precipitated with ethanol, washed thoroughly, and dried in a vacuum drying oven at 30~50 °C to yield EEUG [31]. EEUG samples with different epoxy degrees *E* (mol%) were designated as VEUG–*b*, where *b* represents the value of *E* (mol%). The FTIR spectroscopy and ^1^H NMR spectroscopy results of the EEUG sample prepared at a [HCOOH]:[·H_2_O_2_] = 1:0.5 are presented in Figure 2a and Figure 2b, respectively. As can be seen from Figure 2a, compared with pristine EUG, a characteristic absorption peak attributed to epoxy groups appears at 1262 cm^−1^ in the EEUG spectrum. In addition, Figure 2b shows that a weak proton resonance peak corresponding to epoxy groups emerges at 2.70 ppm in the ^1^H NMR spectrum of EEUG. These results indicate that partial double bonds in the EUG molecular chains have undergone epoxidation successfully [11].

#### 2.2.3. Preparation of Hydroxylated *Eucommia ulmoides* Gum (HEUG)

First, EEUG was prepared with a molar ratio of [H_2_O_2_]/[C=C] = 0.5:1 and [HCOOH]/[·H_2_O_2_] = 1:1. The as-prepared EEUG was dissolved in xylene solution, and the mixture was placed in a constant temperature oil bath, heated up to 110 °C, and mechanically stirred at a rotation speed of 280 rpm until the EEUG was completely dissolved. Subsequently, glacial acetic acid was added to adjust the pH = 3. Different hydroxyl degrees *H* (mol%) were controlled by regulating the reaction time. The reaction product was precipitated with ethanol, washed thoroughly, and dried to obtain HEUG [31]. HEUG samples with different *H* (mol%) were designated as VEUG–*c*, where *c* represents the value of *H* (mol%). The FTIR spectroscopy (Thermo Fisher Scientific, Nicolet iS10, Waltham, MA, USA) and ^1^H NMR spectroscopy (Bruker, Avance NEO 600 MHz, Karlsruhe, Germany) results of the HEUG sample prepared with a reaction time of 7 h are presented in Figure 2a and Figure 2b, respectively. As can be seen from Figure 2a, a distinct carbonyl stretching vibration peak appears at 3417 cm^−1^ in the HEUG spectrum. It can also be observed from Figure 2b that a hydroxyl proton peak and a methylene proton peak adjacent to hydroxyl groups emerge at 3.53 ppm and 3.8 ppm, respectively, in the HEUG spectrum, which indicates the successful preparation of HEUG [11].

### 2.3. Preparation of Modified Asphalt

#### 2.3.1. Preparation of Vulcanized EUG Modified Asphalt (VEMA)

The VEUG prepared above was merely intended to clarify the vulcanization cross-linking mechanism. According to previous research findings, the direct incorporation of VEUG into asphalt would severely impair the low-temperature performance of asphalt, mainly because the direct addition of VEUG to asphalt is not conducive to the formation of cross-linked networks. Thus, the present study adopts the in situ cross-linking method (i.e., cross-linking EUG directly in asphalt) to prepare VEMA. First, EUG was ground into particles with a particle size of approximately 60 mesh, which were then added to MA. The mixture was mechanically stirred at a rotation speed of 350 rpm for 30 min, followed by swelling and curing in an oven at 145 °C for 1 h. The cured blend was taken out and subjected to shearing at 160 °C and 4000 rpm for 45 min using a high-speed emulsifying shearing machine and then placed in the oven again to remove air bubbles, yielding EUG-modified asphalt (EUGMA). Subsequently, sulfur, SA, ZnO, CBS, and IPPD were added sequentially into the as-prepared EUGMA at a dosage of 3.5, 2, 5, 2, 2, and 6 phr, respectively (based on the mass of EUG). The mixture was sheared for 15 min and then subjected to swelling and curing in an oven at 145 °C for 120 min to obtain VEMA, which was designated as VEMA-3.5/6.0.

#### 2.3.2. Preparation of Epoxidized EUG Modified Asphalt (EEMA)

EEUG was also ground into particles with a particle size of approximately 60 mesh and then added into MA at a dosage of 5 wt.% relative to the mass of MA. The mixture was mechanically stirred at a rotation speed of 350 rpm for 30 min to achieve the uniform dispersion of EEUG. Subsequently, the mixture was sheared at 160 °C and 4000 rpm for 45 min using a high-speed emulsifying shearing machine to obtain EEMA. The modified asphalt samples prepared using EEUG with different epoxy degrees *E* (mol%) were designated as EEMA–*d*, where *d* represents the value of *E* (mol%).

#### 2.3.3. Preparation of Hydroxylated EUG Modified Asphalt (HEMA)

Following the same preparation method as that for EEMA, HEMA can be obtained simply by adjusting the shearing temperature to 145 °C. The modified asphalt samples prepared using EEUG with different hydroxyl degrees *H* (mol%) were designated as HEMA-*e*, where *e* represents the value of *H* (mol%).

### 2.4. Design of Experimental Scheme

#### 2.4.1. Structural Characterization Tests of Chemically Modified EUG

(1) Proton nuclear magnetic resonance spectroscopy (^1^H NMR) tests: A Bruker Avance NEO 600 MHz NMR spectrometer from Germany was used, with deuterated chloroform as the solvent at a concentration of 5 mg/mL and tetramethylsilane as the internal standard; the number of scans was 16. The epoxidation degree *E* (mol%) and hydroxylation degree *H* (mol%) can be calculated according to Equations (1) and (2), respectively [4]:

(1) $$E(\mathrm{mol}\%)=\frac{A_{2.73}}{A_{2.73}+A_{5.12}}\times 100$$

Hydroxyls are divided into *α*- and *β*-hydroxyl:

(2) $$H(\mathrm{mol}\%)=\frac{A_{2.73}}{A_{2.73}+A_{5.12}+A_{3.25–4.10}+A_{4.70–4.90}}\times 100\;H_{\alpha }(\mathrm{mol}\%)=\frac{A_{4.70–4.90}}{A_{2.73}+A_{5.12}+A_{3.25–4.10}+A_{4.70–4.90}}\times 100\;H_{\beta }(\mathrm{mol}\%)=\frac{A_{3.25–4.10}}{A_{2.73}+A_{5.12}+A_{3.25–4.10}+A_{4.70–4.90}}\times 100$$

where *A*_2.73_ is the peak integral area of the epoxy group, *A*_5.12_ is the peak integral area of the unsaturated C=C bond, and *A*_4.70–4.90_ and *A*_3.25–4.10_ are the peak integral areas of *α*- and *β*-hydroxyl, respectively.

(2) Fourier transform infrared spectroscopy (FTIR) tests: The modifiers were tested using a ThermoFisher Nicolet iS10 FTIR spectrometer from USA, with a resolution of 4 cm^−1^, a scanning number of 32 times, and a wavenumber test range of 4000~400 cm^−1^. The functional group index was calculated according to Equation (3) [12]:

(3) $$I_{\mathrm{Characteristic}\;\mathrm{functional}\;\mathrm{group}}=\frac{{\sum A}_{\mathrm{Characteristic}\;\mathrm{peak}}}{{\sum A}_{500\sim {3100\;\mathrm{cm}}^{-1}}\;}$$

(3) Gel permeation chromatography (GPC) tests: The samples were dissolved in tetrahydrofuran eluent and then filtered through a 0.45 mm filter. The test was conducted with an injection volume of 0.05 mL, a flow rate of 1.0 mL/min, and a temperature of 40 °C for a duration of 40 min.

#### 2.4.2. Crystallization Property Tests of Chemically Modified EUG

(1) Differential scanning calorimetry (DSC) tests: Approximately 4 mg of the sample was placed in an aluminum crucible. The test was conducted under nitrogen protection, with a heating and cooling rate set at 10 °C/min and a temperature range of −80~150 °C. The crystallinity (*X*_c_*) was calculated according to Equation (4) [8]:

(4) $$X_{c}^{*}=\frac{\Delta H_{f}}{\Delta H_{f100\%}}\times 100$$

where Δ*H*_f_ is the melting enthalpy of the sample obtained from the DSC curve; Δ*H*_f100%_ is the melting enthalpy of 100% crystalline EUG, with a value of 125.9 J/mg.

(2) Wide-angle X-ray diffraction (WAXD) tests: The samples were cut into rectangular sheets with dimensions of 50 × 10 × 1 mm using a cutting knife, and the tests were performed at room temperature. The sample crystallinity (*X*_c_) was calculated according to Equation (5) [11]:

(5) $$X_{c}=\frac{A_{c}}{A_{c}+A_{\alpha }}\times 100$$

where *A*_c_ and *A_α_* represent the areas of the crystalline peaks and amorphous regions in the WAXD patterns, respectively.

#### 2.4.3. Thermal Stability and Hydrophilicity Tests of Chemically Modified EUG

(1) Thermogravimetric analysis (TGA) tests: The test was conducted in a nitrogen atmosphere, with a temperature range of 25~800 °C and a heating rate of 10 °C/min.
(2) Contact angle tests: The contact angle of modified EUG was measured using the sessile drop method. The sample thickness was 0.5~1 mm. Before the test, the sample surface was purged with nitrogen for 1~2 min to avoid dust interference. The temperature was controlled at 25 ± 1 °C, deionized water was selected as the test liquid, and the droplet volume was controlled at 2~5 μL. After the droplet fell onto the sample surface, it was allowed to stand for 1~3 s to stabilize its morphology, and then the contact angle was measured and recorded. The average value of the test results from 5 groups of samples was taken as the final result.

#### 2.4.4. Mechanical Properties Tests of Chemically Modified EUG

(1) Hardness test: The hardness test was performed using an SS-8010D hardness tester in accordance with the specification of GB/T 1698-2003. The specimens were smooth and flat thin plates with dimensions of 50 × 50 mm and a thickness of 4 mm. The arithmetic mean value of 10 valid tests was taken as the final hardness value.
(2) Impact test: The impact test was conducted using an SS-3700CZ pendulum impact tester following the standard of GB/T 1843-2008. The test specimens were standard splines with V-shaped notches, and the pendulum energy was set at 2.75 J. The arithmetic mean value of the test results from 5 specimens was adopted as the final data.
(3) Tensile test: The tensile test was carried out using an SS-8600 electronic universal tensile testing machine in compliance with the regulation of GB/T 528-1998. The initial gauge length was 50 mm, the test environment was set at room temperature (25 °C), and the tensile rate was 50 mm/min. The arithmetic mean value of the test results from 5 specimens was taken as the final result.
(4) Tear test: The tear test was performed with an SS-8600 electronic universal tensile testing machine according to the specification of GB/T 529-2008. The test specimens were of the right-angle type (in accordance with GB 530-81) with a thickness of 2 mm. The arithmetic mean value of the test results from 5 specimens was used as the final data.
(5) Compression test: The compression test was conducted using an SS-8600HD high and low temperature electronic universal testing machine following the standard of GB/T 7757-2014. The arithmetic mean value of the test results from 5 specimens was taken as the final result.

#### 2.4.5. Design of Experimental Scheme for Chemically Modified EUG Modified Asphalt

(1) Temperature sweep test: The temperature sweep test was conducted using a dynamic shear rheometer (DSR). Parallel plates with a diameter of 25 mm were selected, and the plate gap was set at 1 mm. The test temperature range was 46~76 °C with a temperature interval of 6 °C, and the loading frequency was 10 rad/s.
(2) Multiple stress creep and recovery (MSCR) test: The MSCR test was performed using a DSR. Parallel plates with a diameter of 25 mm were adopted, and the plate gap was set at 1 mm. The test temperature was 64 °C. In the first stage, a stress of 0.1 kPa was applied to simulate light traffic loads; in the second stage, a stress of 3.2 kPa was used to simulate heavy traffic loads. A total of 30 loading cycles were applied in the experiment, with each cycle consisting of 1 s of creep and 9 s of recovery. The non-recoverable creep compliance (*J*_nr_) can be used to evaluate the resistance of asphalt to permanent deformation, and the elastic recovery rate (*R*) can be used to evaluate the elastic recovery capability of asphalt, which were calculated using Equations (6) and (7), respectively [14]:

(6) $$J_{\mathrm{nr}}=\frac{1}{10}\sum_{1}^{10}\frac{\varepsilon_{r}-\varepsilon_{0}}{\delta }$$

(7) $$R=\frac{1}{10}\sum_{1}^{10}\frac{\left(\varepsilon_{c}-\varepsilon_{0})-(\varepsilon_{r}-\varepsilon_{0}\right)}{\varepsilon_{c}-\varepsilon_{0}}$$

where *ε*_c_ is the peak strain, *ε*_r_ is the unrecovered strain, *ε*_0_ is the initial strain, and *δ* is the stress.

(3) Low-temperature bending beam rheometer (BBR) test: The asphalt was heated to a molten state in an oven at 145 °C for 30 min and then poured into molding dies and cooled for 45 min. The asphalt samples were placed in a water bath at −5 ± 5 °C for 10 min before being demolded. The test temperature range was −12 °C~−24 °C with a temperature gradient of 3 °C. The final results were taken as the average value of three groups of specimens.
(4) Atomic force microscopy (AFM) test: The modified asphalt was tested using a Bruker Dimension ICON AFM in QNM mode. The scanning rate was set at 1 Hz, the test temperature was 25 °C, the scanning area was 15 μm × 15 μm, and the resolution was 512 × 512.

## 3. Results and Discussion

### 3.1. Property Analysis of Chemically Modified EUG

#### 3.1.1. Effects of Reaction Conditions on *E* (mol%) and *H* (mol%)

**Figure 3:**
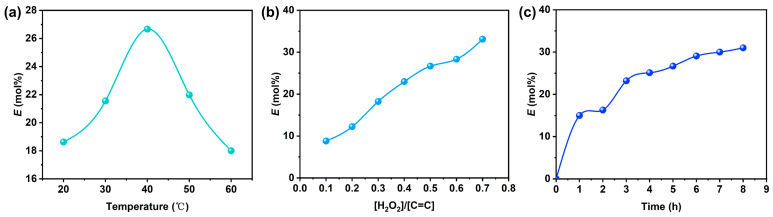
Effects of (**a**) reaction temperature ([H_2_O_2_]/[C=C] = 0.5, 5 h), (**b**) H_2_O_2_ dosage (40 °C, 5 h) and (**c**) reaction time ([H_2_O_2_]/[C=C] = 0.5, 40 °C) on *E* (mol%).

During the preparation of EEUG, EEUG was obtained via the epoxidation of EUG by in situ generating peroxy acid through adding formic acid and H_2_O_2_ solution into xylene solution. The reaction temperature, [H_2_O_2_]/[C=C] ratio, and reaction time exert significant effects on *E* (mol%), as shown in Figure 3. It can be seen from Figure 3 that the *E* (mol%) first increased and then decreased with the elevation in the temperature, reaching the maximum value at 40 °C. This is because when the temperature was below 40 °C, a large number of epoxy groups were generated, leading to a rapid increase in *E* (mol%). When the temperature exceeded 40 °C, the epoxy groups would undergo ring-opening reaction to form furan structures or induce molecular chain cross-linking, resulting in gelation, which caused the decrease in *E* (mol%) with the further rise in temperature. Under the reaction conditions of 40 °C and 5 h, the *E* (mol%) increased with the increase in the H_2_O_2_ dosage, indicating that *E* (mol%) could be adjusted by controlling the dosage of H_2_O_2_. Under the conditions of [H_2_O_2_]/[C=C] = 0.5 and 40 °C, the *E* (mol%) increased with the extension of the reaction time, and the growth rate of *E* (mol%) tended to be stable after 3 h of reaction due to the consumption of H_2_O_2_ [31].

Epoxides possess significant ring strain due to their three-membered ring structure and thus can undergo ring-opening reactions. As nucleophiles, acids can attack epoxy groups to generate hydroxyl-functionalized products. In this study, HEUG was prepared via the ring-opening reaction of EEUG with *E* (mol%) = 26.67% and glacial acetic acid under catalyst-free conditions. The effects of the reaction temperature, pH value, and reaction time on *H* (mol%) were investigated, and the results are shown in Table 4. With the reaction temperature and pH kept constant, *H* (mol%) increased with the extension of the reaction time; after 5.5 h of reaction, the growth rate of *H* (mol%) slowed down, due to the presence of steric hindrance and ortho-substituents. Increasing the reaction temperature was conducive to the conversion of epoxy groups to hydroxyl groups, while an increase in pH significantly inhibited the ring-opening reaction of epoxy groups [9].

#### 3.1.2. Structural Analysis of Chemically Modified EUG

**Figure 4:**
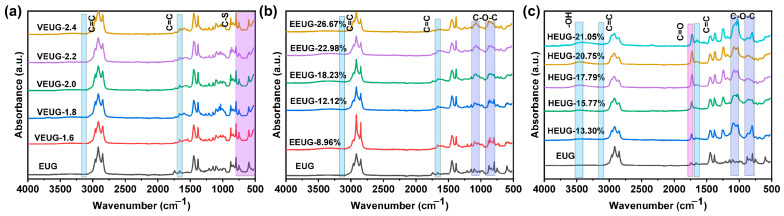
FTIR results of chemically modified EUG: (**a**) VEUG; (**b**) EEUG; (**c**) HEUG.

The FTIR results of various chemically modified EUG samples are presented in Figure 4. As can be seen from Figure 4, the absorption peaks of EUG at 3050 cm^−1^ and 1666 cm^−1^ are attributed to the stretching vibration of unsaturated C=C double bonds in EUG, and these two peaks are the characteristic signals of unsaturated C=C double bonds in the EUG molecular chain [32]. It can be observed from Figure 4a that, with the increase in the sulfur dosage, the characteristic peaks of C=C double bonds of VEUG at 3050 cm^−1^ and 1666 cm^−1^ gradually weaken, indicating that the C=C double bonds are gradually consumed by participating in the vulcanization reaction. Meanwhile, the absorption peaks in the range of 500~800 cm^−1^ become increasingly sharp with the rise in the sulfur dosage, which correspond to the stretching vibration signals of C–S bonds generated by the reaction between sulfur and C=C double bonds, and are the characteristic vibration peaks unique to VEUG. In addition, the shape and intensity of the stretching vibration peak of saturated alkyl C−H at 2920 cm^−1^ remain basically unchanged, demonstrating that only the cross-linking reaction between C=C double bonds and sulfur occurs during the vulcanization process [33]. As shown in Figure 4b, after the epoxidation of EUG, the intensity of C=C double bond peaks decreases, and new stretching vibration peaks of epoxy groups (C−O−C) appear at 878 cm^−1^ and 1250 cm^−1^. Moreover, the characteristic peaks of C−O−C gradually strengthen with the increase in *E* (mol%), which confirms the successful occurrence of the epoxidation reaction [34]. It can also be seen from Figure 4c that the stretching vibration peak intensity of C−O−C at 878 cm^−1^ and 1250 cm^−1^ decreases after the ring-opening grafting of −OH groups. With the increase in *H* (mol%), the peak intensity of −OH at 3476 cm^−1^ increases continuously, while the characteristic peaks of C=C double bonds at 3050 cm^−1^ and 1666 cm^−1^ basically disappear. These phenomena indicate that both C=C double bonds and epoxy groups are continuously consumed by participating in the ring-opening reaction, thus verifying the successful realization of the ring-opening reaction.

**Figure 5:**
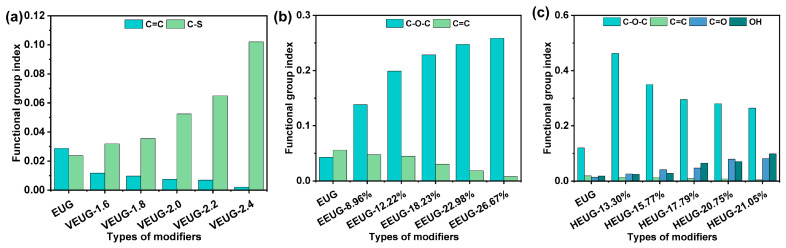
Changes in chemical functional group indices of modified EUG: (**a**) VEUG; (**b**) EEUG; (**c**) HEUG.

**Figure 6:**
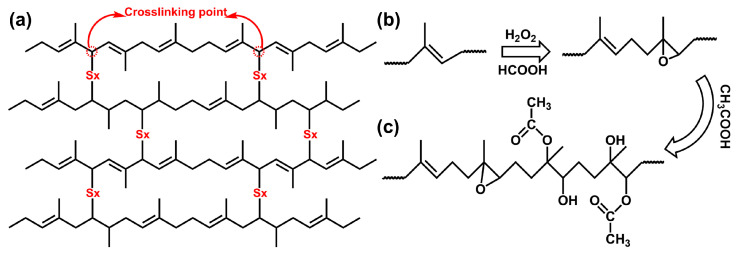
Schematic diagram of the reaction process of modified EUG: (**a**) VEUG; (**b**) EEUG; (**c**) HEUG.

The changes in the chemical functional group indices of various modified EUG samples are shown in Figure 5. As can be seen from Figure 5a, *I*_C=C_ decreases and *I*_C–S_ increases after vulcanization. With the increase in the sulfur dosage, *I*_C=C_ decreases continuously while *I*_C−S_ increases continuously, indicating that sulfur reacts with C=C double bonds during the vulcanization process, consuming C=C double bonds and generating C−S bonds. The reaction process of VEUG is illustrated in Figure 6a. After vulcanization, the independently distributed EUG molecular chains are connected by sulfur bridge bonds (–S_x_–). As the sulfur dosage increases, the vulcanization reaction of EUG becomes more complete, and the cross-linking degree becomes deeper, realizing the transition of the molecular structure from a linear unsaturated structure to a network cross-linked structure. It can also be observed from Figure 5b that *I*_C−O−C_ increases and *I*_C=C_ decreases after the epoxidation of EUG. With the increase in *E* (mol%), *I*_C−O−C_ increases continuously, and *I*_C=C_ decreases continuously, demonstrating that epoxidation is mainly achieved by the in situ generated peroxy acid from H_2_O_2_ and formic acid attacking the C=C double bonds in the main chain of EUG to form epoxy groups. Moreover, as *E* (mol%) increases, the C=C double bonds are continuously destroyed, and the content of the epoxy groups increases. The corresponding reaction process is shown in Figure 6b. It can also be found from Figure 5c that, compared with pristine EUG, *I*_C−O−C_, *I*_C=O,_ and *I*_−OH_ increase, while *I*_C=C_ decreases, after the hydroxylation of EUG. With the increase in *H* (mol%), *I*_C−O−C_ and *I*_C=C_ decrease continuously, whereas *I*_C=O_ and *I*_–OH_ increase continuously. This indicates that during the hydroxylation process, glacial acetic acid attacks both epoxy groups and C=C double bonds; the epoxy groups undergo ring-opening reaction to graft −OH groups. As *H* (mol%) increases, the ring-opening grafting reaction of EEUG is continuously enhanced. The increase in *I*_C=O_ may be attributed to the fact that the intermediate products in the reaction process contain unsaturated double bonds, which are oxidized by oxygen in the air or oxidizing substances in the system under high temperature and acidic conditions. The hydroxylation reaction process is presented in Figure 6c.

Since VEUG forms an internal cross-linked network during vulcanization and cannot be dissolved in organic solvents, only the molecular weight distribution of EEUG and HEUG was analyzed, as shown in Table 5. It can be seen from Table 5 that, after epoxidation, the number-average molecular weight (*M*_n_), weight-average molecular weight (*M*_w_), and polydispersity index (PDI) of EEUG all decrease. Moreover, with the increase in *E* (mol%), *M*_n_ and *M*_w_ decrease continuously, and the molecular weight distribution becomes more uniform. This is attributed to two reasons. On the one hand, peroxy acid attacks the molecular backbone of EUG, leading to the cleavage of C=C double bonds and the formation of more small molecules. As the dosages of H_2_O_2_ and HCOOH increase, the reaction with EUG becomes more intense, resulting in a lower molecular weight. On the other hand, the epoxy groups generated under acidic conditions may further undergo ring-opening reactions accompanied by the cleavage of chemical bonds, breaking the long polymer chains into smaller fragments. The higher the *E* (mol%), the more these potential molecular breakage points exist, leading to lower molecular weight and more uniform molecular weight distribution. Compared with EEUG, HEUG has smaller *M*_n_ and *M*_w_ and a more uniform molecular weight distribution. Additionally, with the increase in *H* (mol%), *M*_n_ and *M*_w_ decrease continuously, and the molecular weight distribution becomes even more uniform. This is because under acidic conditions, the epoxy groups of EEUG undergo cleavage while being grafted with −OH groups via ring-opening reaction. As the reaction time increases, *H* (mol%) rises continuously, longer molecular chains break, and the molecular weight decreases accordingly. Furthermore, the longer molecular chains tend to break preferentially during the reaction, which makes the molecular weight distribution more uniform, and PDI decreases gradually [35].

#### 3.1.3. Crystallization Properties Analysis of Chemically Modified EUG

**Figure 7:**
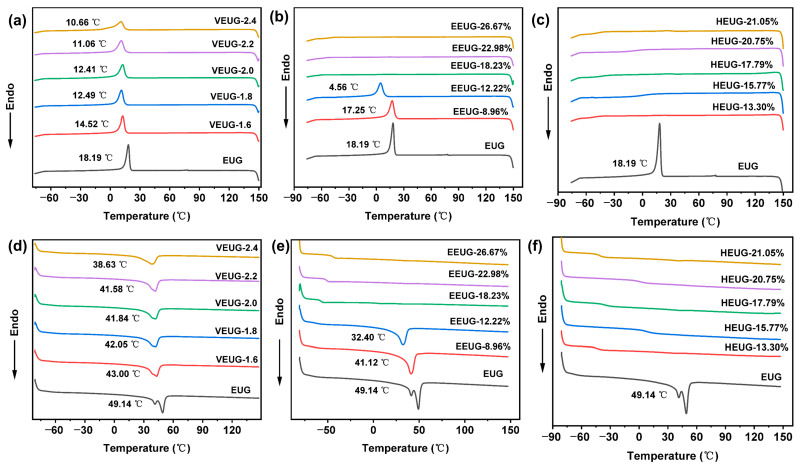
DSC curves of modified EUG: (**a**) DSC cooling curve of VEUG; (**b**) DSC cooling curve of EEUG; (**c**) DSC cooling curve of HEUG; (**d**) DSC heating curve of VEUG; (**e**) DSC heating curve of EEUG; (**f**) DSC heating curve of HEUG.

The melting–crystallization behavior of different modified EUG samples can be obtained from the DSC curves, with the results shown in Figure 7 and Table 6. As can be seen from Figure 7a,d, EUG exhibits a sharp and high crystallization peak in the temperature range of 15~25 °C, indicating that EUG is prone to crystallization at room temperature, with the maximum crystallization peak temperature being 18.19 °C. With the increase in the vulcanization degree, both the crystallization peak and melting peak continuously shift toward lower temperatures, and the crystallinity (*X*_c_*) decreases. This is because after vulcanization, EUG molecular chains are cross-linked and immobilized by sulfur bridge bonds, which inhibits the regular arrangement of EUG molecular chains, making it difficult for VEUG to crystallize and thus leading to a decrease in *X*_c_*. The internal order and stability of VEUG are reduced; so, its crystal structure can be destroyed at a lower temperature, resulting in the decrease in the melting temperature (*T*_m_) and enthalpy value (Δ*H*). Meanwhile, the disordered and loose state of molecular chains enables them to rearrange and crystallize at a lower temperature, causing the crystallization temperature (*T*_c_) to decrease. Furthermore, as the vulcanization degree increases, the cross-linking degree of EUG molecular chains becomes deeper, and the ability to inhibit their regular arrangement is continuously enhanced [36]. With the increase in the vulcanization degree, the glass transition temperature (*T*_g_) increases, indicating the transition of the material from rigid plasticity to elasticity. This is because as the vulcanization degree increases, the cross-linked network is continuously strengthened, which strongly restricts the movement of molecular chains, and the chain segments in the amorphous phase need higher energy to overcome this restriction.

As can be seen from Figure 7b,e, with the increase in *E* (mol%), the crystallization and melting peaks of EEUG shift toward lower temperatures, while the peak area continuously decreases, the peaks broaden and eventually disappear, and *X*_c_* decreases accordingly. When *E* (mol%) > 18.23%, the crystallization and melting peaks of EEUG disappear, indicating that EEUG loses its crystallinity completely. This is because the introduction of epoxy groups converts a large number of non-rotatable C=C double bonds into rotatable C−C single bonds, thereby reducing the symmetry and orderliness of molecular chains, which results in a decline or even complete loss of crystallization ability. Although crystal nuclei can be formed and even small crystals can be generated at the cooling rate applied in the DSC test, the crystals have no time to grow. During the subsequent heating process, these crystal nuclei or small crystals undergo secondary crystallization and grow larger, leading to the decrease in Δ*H*, *T*_m,_ and *T*_c_. With the increase in *E* (mol%), *T*_g_ increases monotonically, and EEUG gradually transforms into an elastic material. This is because the introduction of polar functional groups destroys the crystal structure and reduces the stiffness of molecular chain segments.

It can also be found from Figure 7c,f that HEUG shows no obvious crystallization peaks or melting peaks. This is because HEUG is prepared by ring-opening grafting of −OH groups based on EEUG with *E* (mol%) = 26.67%. When *E* (mol%) > 18.23%, the epoxy groups have already destroyed the regularity and symmetry of EUG molecular chains; after grafting with −OH groups, the regularity and symmetry of the molecular chains are further impaired, resulting in the complete loss of crystallization ability. In addition, the introduction of −OH groups further reduces the symmetry and orderliness of the molecular chains, decreases the stiffness of the molecular chain segments, and thus leads to an increase in *T*_g_ [7,37].

**Figure 8:**
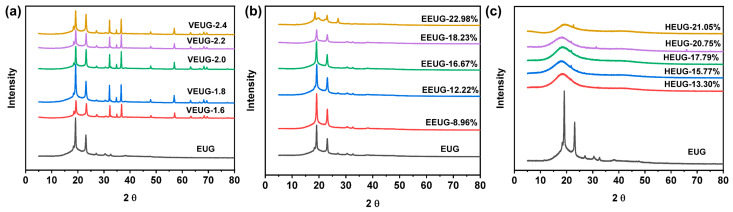
WAXD curves of modified EUG: (**a**) VEUG; (**b**) EEUG; (**c**) HEUG.

To further verify the crystallization properties of modified EUG, it was characterized by WAXD, with the results shown in Figure 8. As can be seen from Figure 8, EUG exhibits distinct sharp peaks at 2θ = 18.7° and 2θ = 22.6°, indicating that the EUG used has a coexisting crystal structure of both *α* and *β* crystal forms, dominated by the *α* crystal form. It can be observed from Figure 8a that, with the increase in the vulcanization degree, the peak shape remains unchanged, the intensity of *α* and *β* crystallization peaks decreases, the peaks broaden, and multiple secondary peaks appear at 2θ > 30°. This demonstrates that the vulcanization reaction does not alter the basic type of crystal structure; instead, the cross-linked network formed inside EUG hinders the ordered arrangement of molecular chains, thereby inhibiting the crystallization of EUG. The higher the vulcanization degree, the stronger the inhibitory effect. In addition, the vulcanization reaction may also generate new, smaller, and imperfectly ordered structures. As shown in Figure 8b, with the increase in *E* (mol%), the shape of *α* and *β* crystallization peaks remains unchanged, while the peak intensity decreases, and the peaks broaden. This indicates that the epoxy groups introduced into the EUG molecular chains after epoxidation destroy the regularity of EUG molecular chains, reducing their ability to form crystal structures. When *E* (mol%) = 22.98%, the intensity of the *α* crystallization peak decreases significantly, the peak broadens, and a strong secondary peak forms at 2θ = 27.2°. This is because under the action of high *E* (mol%), the epoxy groups destroy the regularity of EUG molecular chains, making it difficult to form ordered crystal structures. It can also be found from Figure 8c that, after the hydroxylation of EUG, the *β* crystallization peak is greatly weakened or even disappears, and the intensity of the *α* crystallization peak decreases, and the peak broadens with the increase in *H* (mol%). This is because the introduction of −OH groups further destroys the regularity and symmetry of EUG molecular chains based on epoxidation. In addition, the strong hydrogen bond network formed between −OH groups strongly limits the mobility of chain segments and the rate of conformational adjustment, making it difficult to form *β*–type crystals of the metastable phase [9,38].

**Figure 9:**
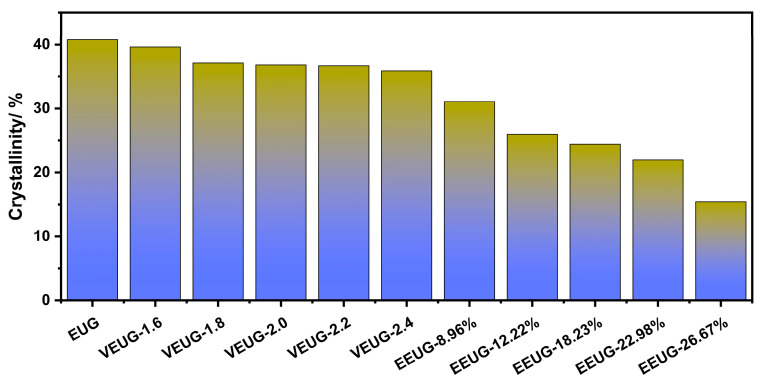
Changes in crystallinity of modified EUG.

The *X*_c_ values calculated via WAXD are shown in Figure 9. *X*_c_* and *X*_c_ exhibit the same variation trend: with the increase in the vulcanization degree, the decrease in the crystallinity of VEUG is not significant; as *E* (mol%) increases, the *X*_c_ of EEUG decreases sharply, and HEUG shows no crystallinity at all. This indicates that both epoxidation and hydroxylation can destroy the regularity and symmetry of EUG molecular chains. With the deepening of the modification degree, the crystallinity of EUG molecular chains decreases sharply until it disappears completely.

#### 3.1.4. Thermal Stability and Hydrophilicity Analysis of Chemically Modified EUG

**Figure 10:**
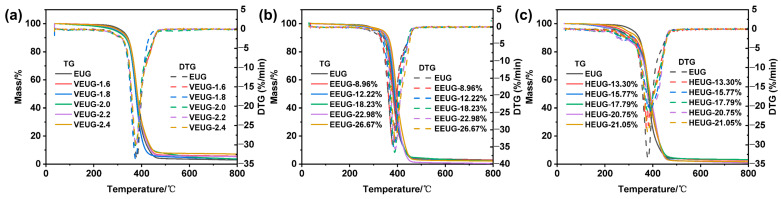
TG and DTG curves of modified EUG: (**a**) VEUG; (**b**) EEUG; (**c**) HEUG.

The TGA curves of various modified EUG samples are shown in Figure 10, and the corresponding characteristic thermal parameters, including the initial decomposition temperature (T_onset_), 5% weight loss temperature (T_5%_), 10% weight loss temperature (T_10%_), maximum decomposition temperature (T_max_), and char yield (CY), are summarized in Table 7. For all EUG samples, the weight loss in the temperature range of 25~200 °C is mainly attributed to the vaporization of internal bound water, while the dominant thermal weight loss occurs at 200~500 °C, which originates from the degradation of polymer molecular chains.

Compared with pristine EUG, VEUG exhibits lower T_onset_ and T_5%_ but higher CY. This is because sulfur bridge bonds formed inside EUG after vulcanization have much lower bond energy than C−C single bonds and C=C double bonds. Upon heating, sulfur bridge bonds break preferentially and generate active free radicals, which accelerate the cleavage of adjacent main chains, thus reducing the overall T_onset_ of VEUG and leading to its poor initial thermal stability. With the increase in the vulcanization degree, T_onset_ and T_5%_ decrease continuously, T_10%_ and T_max_ show negligible changes, and CY increases steadily. The underlying mechanism is that a higher vulcanization degree further deteriorates the initial thermal stability and lowers T_5%_, while the three-dimensional cross-linked network formed by vulcanization inhibits the volatilization of low-molecular-weight substances; so, CY rises with the enhancement of the cross-linked network.

In contrast to pristine EUG, EEUG has higher T_onset_, T_5_%, T_10%_ and T_max_. This is because the introduction of epoxy groups enhances intermolecular forces, and more energy is required to destroy the movement of chain segments during pyrolysis, thus improving the thermal stability. When the epoxy content *E* (mol%) is lower than 18.23%, T_onset_, T_10%_, and T_max_ increase slightly with the rise in *E* (mol%). When *E* (mol%) reaches 26.67%, T_onset_, T_5%_ and T_10%_ increase significantly. At low epoxy content, the epoxy groups are relatively uniformly and sparsely distributed on EUG molecular chains; the number of epoxy groups is insufficient to cause significant changes in the aggregated structure, and the cross-linking degree of molecular chains is low, leading to a mild and gradual improvement in thermal stability, manifested as a slight synchronous increase in thermal decomposition temperature indices. When *E* (mol%) increases to 26.67%, a large number of epoxy groups severely hinder the free rotation of the main chain, making the conformational transition of the entire molecular chain extremely difficult. A higher energy barrier needs to be overcome during decomposition, which strongly improves the thermal stability of EEUG.

Compared with pristine EUG, the DTG curve of HEUG shows a lower peak intensity and broader peak shape. Pristine EUG is a polymer with a highly regular structure, high crystallinity, and a single molecular chain structure. During heating decomposition, once the molecular chain breaks at a weak point, the generated free radicals will rapidly propagate along the regular molecular chain, leading to rapid and concentrated breakage of molecular chains. As a result, the weight loss of pristine EUG occurs in a very narrow temperature range, and its DTG curve presents a high-intensity sharp peak. After hydroxylation, the crystallinity of HEUG decreases, and the amorphous region increases. Molecular chains in the amorphous region are loosely packed with various entanglement forms, which widens the originally concentrated decomposition temperature range, thus resulting in a lower peak intensity and broader peak shape in the DTG curve. Except for T_max_, all thermal decomposition indices of HEUG are lower than those of pristine EUG. This is because the introduction of thermally unstable −OH groups reduces the thermal stability of HEUG and simultaneously promotes the formation of more volatile products, thus reducing the generation of stable solid char yield. With the increase in the hydroxyl content *H* (mol%), T_onset_ and T_5%_ increase, which is ascribed to the formation of a relatively stable hydrogen bond network between −OH groups, leading to the continuous enhancement of the resistance to initial thermal decomposition.

**Figure 11:**
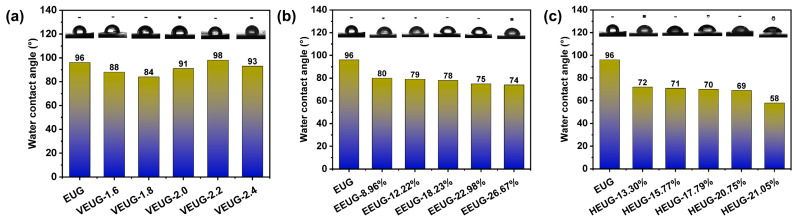
Water contact angles of modified EUG: (**a**) VEUG; (**b**) EEUG; (**c**) HEUG.

The hydrophilicity of modified EUG was characterized by water contact angle measurement, with the results shown in Figure 11. As can be seen from Figure 11a, with the increase in the vulcanization degree, the contact angle shows no obvious regular pattern, and the contact angle of VEUG is similar to that of EUG, indicating that vulcanization has no significant effect on the hydrophilicity of EUG. It can be observed from Figure 11b that the contact angle of EUG decreases after epoxidation, and the contact angle of EEUG decreases continuously with the increase in *E* (mol%). This demonstrates that EEUG has good hydrophilicity, and the hydrophilicity is continuously enhanced with the increase in *E* (mol%). The reason is that strong polar epoxy groups are introduced onto the non-polar EUG molecular chains after epoxidation, and these strongly polar epoxy functional groups have good hydrophilicity. The higher the *E* (mol%), the higher the site density of strongly polar epoxy functional groups on the molecular chains, and the stronger the hydrophilicity. It can also be found from Figure 11c that the contact angle of HEUG is smaller than that of EEUG, and the contact angle further decreases with the increase in *H* (mol%), leading to enhanced hydrophilicity. This is because hydroxylation introduces −OH groups based on epoxidation, and the −OH groups can interact with water molecules through hydrogen bonds, enabling water molecules to combine more closely with HEUG. With the increase in *H* (mol%), more −OH groups can bind to water molecules, resulting in stronger hydrophilicity.

#### 3.1.5. Mechanical Properties Analysis of Chemically Modified EUG

**Figure 12:**
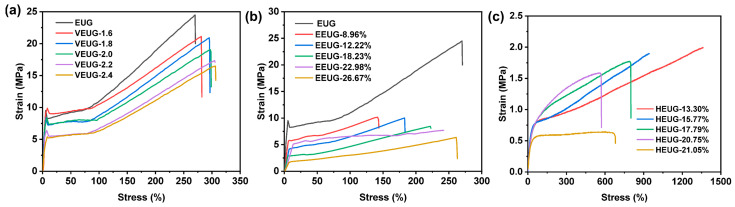
Stress–strain curves of modified EUG: (**a**) VEUG; (**b**) EEUG; (**c**) HEUG.

The tensile test results of modified EUG are shown in Figure 12, and the respective mechanical indices are presented in Table 8. As can be seen from Figure 12a, both EUG and VEUG exhibit the typical stress–strain curves of crystalline polymers, featuring distinct stress yielding and strain hardening characteristics. At the initial strain stage, the EUG curve shows the largest slope, corresponding to a higher modulus and maximum hardness. With the increase in the vulcanization degree, the slope decreases, the elastic modulus becomes smaller, and VEUG turns softer. Meanwhile, the tear strength (*T*_s_), tensile strength (*σ*), and elongation at break (*ε*) of VEUG are continuously improved. This is because as the vulcanization degree increases, the crystallinity of VEUG decreases, the crystalline regions are reduced, and VEUG gradually transforms into a structure dominated by amorphous regions. Within the amorphous regions, the mobility of molecular chain segments is enhanced, enabling them to rotate, stretch, and retract more freely. This structural transformation is macroscopically manifested as the gradual softening of the material and the reduction in modulus, as well as the material’s primary response mechanism of reversible elastic deformation under stress, thus exhibiting higher elasticity, higher deformation capacity, and more significant resilience. When the strain exceeds 100% and enters the high-strain region, an obvious hardening phenomenon occurs; with the increase in the vulcanization degree, the curve tends to be gentler, and the hardening phenomenon is gradually weakened.

As can be seen from Figure 12b, when *E* (mol%) < 22.98%, EEUG exhibits slight stress yielding and strain hardening behaviors. When *E* (mol%) = 26.67%, EEUG shows the characteristic of high elasticity, with the curve rising smoothly and no obvious stress yield point observed. With the increase in *E* (mol%), the initial slope of the curve decreases, the stiffness of EEUG reduces, the elasticity increases, and *T*_s_, *σ*, and *σ*_100%_ decrease continuously, while *ε* increases steadily. This can be attributed to the fact that the introduction of epoxy groups destroys the regularity and symmetry of molecular chains, inhibits the crystallization ability of EUG, makes it gradually transform into an amorphous phase, and thus initiates the transition from rigid plasticity to high elasticity.

As shown in Figure 12c, compared with EUG, the stress yield point of HEUG becomes ambiguous or even disappears completely, with no obvious plastic stretching stage observed. The strain hardening phenomenon of EUG is completely inhibited, the curve rises gently, and *σ* and hardness decrease significantly, while *ε* increases strongly. When *H* (mol%) < 20.75%, with the increase in *H* (mol%), *σ*, *ε*, and the hardness decrease continuously, whereas *σ*_200%_ and *σ*_300%_ increase steadily. This is because the introduction of −OH groups severely destroys the regularity of molecular chains and strongly impairs their crystallization ability. In addition, hydrogen bond networks can be formed between −OH groups; these hydrogen bonds can continuously break and recombine under external forces, which provides certain strength for the material while further inhibiting the crystallization behavior, thus leading to its transition from rigid elasticity to soft elasticity. When *H* (mol%) = 21.05%, HEUG is extremely softened, and the stress–strain curve enters a gentle plateau stage in the strain range of 50–600%, generating large deformation under low stress. *σ* is slightly higher than that of HEUG with *H* (mol%) = 20.75%, while *ε*, *σ*_100%_, *σ*_200%_, *σ*_300%_ and the hardness are lower than those of HEUG with *H* (mol%) = 20.75%. This may be due to the formation of hydrogen bond network structures inside HEUG at high *H* (mol%), where crystallization is greatly inhibited, and the mechanical behavior is mainly determined by amorphous chain segment motion and hydrogen bond networks, exhibiting the characteristics of softness, high elasticity, and large deformation [39].

**Figure 13:**
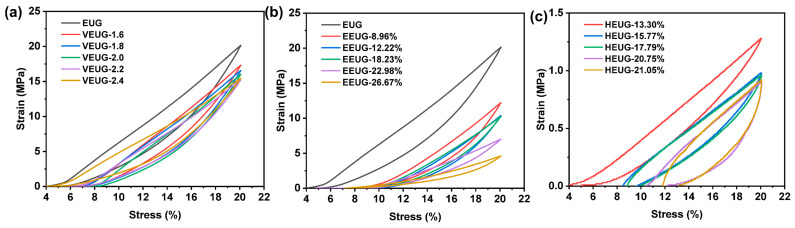
Compression–recovery curves of modified EUG: (**a**) VEUG; (**b**) EEUG; (**c**) HEUG.

Figure 13 shows the compression test results of the modified EUG. As can be seen from Figure 13a, the cyclic stress–strain curve of EUG exhibits a large hysteresis loop and slight permanent deformation, showing obvious plastic behavior. After vulcanization, the hysteresis loop becomes smaller, and it shifts downward with the increase in the vulcanization degree. This indicates that VEUG gradually transitions from plasticity to elasticity, as the vulcanization degree increases. This is because with the increase in the vulcanization degree, the crystallinity decreases, and the independently distributed EUG molecular chains are connected into a whole through sulfur bridge bonds. Under external force, the EUG molecular chains undergo coordinated movement, which is macroscopically manifested as enhanced elasticity. It can be observed from Figure 13b that, after the epoxidation of EUG, the hysteresis loop is significantly reduced and shifts downward. With the increase in *E* (mol%), the hysteresis loop continues to shrink and shift downward, indicating that EEUG also exhibits good elasticity. This can be attributed to the fact that the introduction of epoxy groups destroys the regularity of EUG molecular chains, leading to a decrease in crystallinity. As shown in Figure 13c, when *H* (mol%) = 13.30%, the hysteresis loop is relatively large, and the slope of the loading–unloading curve is high, with HEUG showing hard elasticity characteristics. This is because at low *H* (mol%), HEUG still contains epoxy groups, which provide a certain degree of stiffness, while the introduction of −OH groups also endows it with good elastic recovery ability. When 15.77% < *H* (mol%) < 17.79%, the hysteresis loop shows no obvious change but shifts downward compared with that at *H* (mol%) = 13.30%. This is due to the increased number of ring-opened epoxy groups; HEUG molecular chains form a network structure through hydrogen bonds, and the hydrogen bonds are in a state of dynamic equilibrium of breaking and recombination under stress, making HEUG a transitional soft elastomer. When *H* (mol%) > 20.75%, the hysteresis loop shows little change but shifts to the right compared with that in the range of 15.77% < *H* (mol%) < 17.79%, with permanent deformation reduced. Dense, uniform, and dynamically reversible strong hydrogen bond network structures are formed between −OH groups, and the molecular chains are fixed in the hydrogen bond networks. Under stress, the hydrogen bonds break and recombine rapidly, which is macroscopically manifested as the characteristics of high resilience and low deformation [8].

### 3.2. Performance Analysis of Asphalt Modified by Chemically Modified EUG

To investigate the effects of modified EUG with different dosages on the high and low temperature performance, morphology, and mechanical properties of asphalt, EEUG with *E* (mol%) = 26.67% and HEUG with *H* (mol%) = 21.05% were selected, respectively, to prepare EEMA and HEMA, with the modifier dosages set as 3 wt.%, 5 wt.% and 7 wt.%. Since directly adding VEUG into asphalt is not conducive to the formation of cross-linked networks, this study adopted the method of cross-linking EUG in asphalt to prepare VEMA. Previous findings have demonstrated that the optimal cross-linked network can be formed when the mass ratio of sulfur/EUG is 3.5/6; thus, this mass ratio was selected for the preparation of VEMA.

#### 3.2.1. Analysis of High-Temperature Deformation Resistance of Modified Asphalt

**Figure 14:**
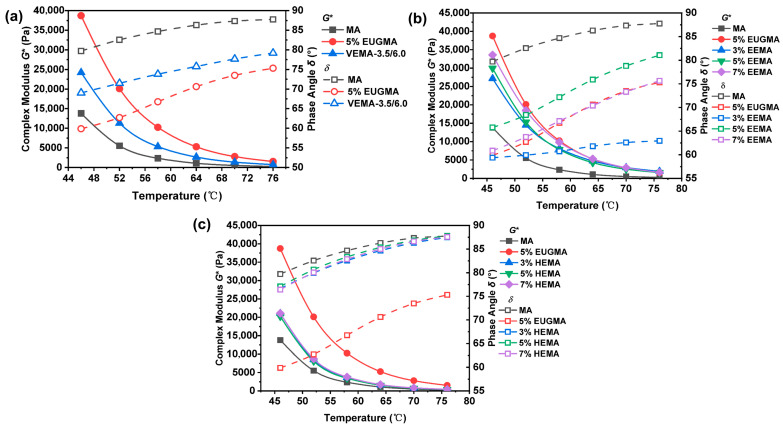
G* and *δ* of various asphalts: (**a**) VEMA; (**b**) EEMA; (**c**) HEMA.

The complex shear modulus (G*) is defined as the ratio of maximum shear stress to shear strain, which characterizes the deformation resistance of materials. The phase angle (*δ*) refers to the phase difference between strain lag and stress in viscoelastic materials, reflecting the relative proportion of viscous and elastic components in the material [2,22]. The rheological test results of various asphalt samples are shown in Figure 14. For all asphalt specimens, G* decreases while δ increases continuously with rising temperature. This trend indicates that elevated temperature drives the conversion of elastic components to viscous components in asphalt, leading to the softening of the asphalt binder and the reduction in its deformation resistance.

As shown in Figure 14a, at the identical test temperature, VEMA presents a higher G* value and lower *δ* value than MA, while its G* is lower, and its *δ* is higher than unmodified EUG modified asphalt (EUGMA). This result demonstrates that VEMA has better deformation resistance than MA but inferior deformation resistance relative to EUGMA. The underlying mechanism is as follows. Pristine EUG has an intrinsically high modulus; so, its direct incorporation endows EUGMA with a high modulus. After vulcanization, the originally dispersed EUG molecules are cross-linked into an integral three-dimensional network, which gives VEMA excellent elastic recovery and deformation resistance (thus outperforming MA). However, the cross-linking modification of EUG weakens the modulus enhancement effect compared with unmodified EUG, resulting in the lower deformation resistance of VEMA than EUGMA.

Figure 14b shows that at the same temperature, EEMA with different epoxidized EUG (EEUG) dosages all exhibit higher G* and lower *δ* than MA, while their G* values are lower than that of EUGMA. This indicates that the incorporation of EEUG improves the high-temperature deformation resistance of MA and increases the proportion of elastic components in the asphalt binder. This is because EEUG is an elastomer, and its introduction into asphalt increases the elastic component content of the system, thus raising the G* of asphalt. In addition, with the increase in the EEUG dosage, the G* value of EEMA increases continuously, revealing that a higher EEUG dosage leads to better high-temperature deformation resistance of modified asphalt. As presented in Figure 14c, at the identical test temperature, HEMA with various hydroxylated EUG (HEUG) dosages all have higher G* and lower *δ* than MA, indicating that the addition of HEUG enhances the high-temperature deformation resistance of MA. This enhancement may be attributed to the hydrogen bond network formed by the −OH of HEUG in the asphalt matrix, which improves the stiffness and modulus of MA. Compared with EUGMA, all HEMA specimens show lower G* and higher *δ*, meaning the deformation resistance of HEMA is weaker than that of EUGMA. This is because hydroxylation modification softens the EUG molecular chain, resulting in a lower modulus enhancement effect of HEUG than pristine EUG. For HEMA with different HEUG dosages, the G* value increases slightly with a narrow variation range, and the *δ* value has no obvious change with the increase in the HEUG dosage. This phenomenon may be ascribed to the following reasons. The HEUG used in this test (*H* (mol%) = 21.05%) already presents an ultra-soft rubber morphology, with limited active sites on its molecular chains. The effective combination between HEUG and polar components in asphalt (such as resins and asphaltenes) via hydrogen bonding and polar adsorption has a threshold limit. A slight increase in HEUG dosage cannot break through this threshold, thus leading to an insignificant change in the deformation resistance of HEMA with rising HEUG content.

**Figure 15:**
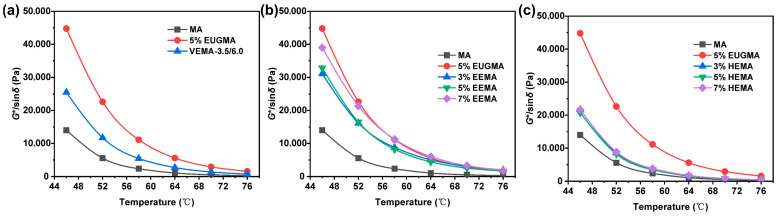
G*/sin*δ* values of various asphalts: (**a**) VEMA; (**b**) EEMA; (**c**) HEMA.

The rutting factor (G*/sin*δ*) can be used to characterize the high-temperature rutting resistance of asphalt; the higher its value, the stronger the rutting resistance [40]. Figure 15 presents the G*/sin*δ* results of different asphalts. It can be observed that G*/sin*δ* decreases with increasing temperature, and the rate of decrease slows down in the temperature range of 64~76 °C. This indicates that when the temperature is between 46 and 64 °C, the elastic components of asphalt decrease rapidly and transform into a viscous flow state. After 64 °C, the asphalt completely exhibits a viscous flow state and loses its deformation resistance. As can be seen from Figure 15a, at the same temperature, the G*/sin*δ* value of VEMA is higher than that of MA but lower than that of EUGMA. This demonstrates that a cross-linked network forms inside VEMA after vulcanization, connecting the independently distributed EUG molecules into an integrated structure [23]. Thus, VEMA exhibits good high-temperature rutting resistance. It can be seen from Figure 15b that, at the same temperature, the G*/sin*δ* value of EEMA is higher than that of MA, indicating enhanced high-temperature shear deformation resistance. This may be because the epoxy groups combine with polar asphalt molecules through covalent bonds, which require higher energy to induce deformation, thus significantly improving the high-temperature deformation resistance of MA compared with the base asphalt [41]. At the same temperature, the G*/sin*δ* value of EEMA increases continuously with the increase in the EEUG dosage. This may be attributed to the fact that a higher dosage provides more contact sites between epoxy groups and polar functional groups of MA, resulting in stronger high-temperature deformation resistance. As shown in Figure 15c, at the same temperature, the G*/sin*δ* value of HEMA is higher than that of MA, with enhanced high-temperature shear deformation resistance. This is probably because the hydrogen bond network formed between −OH groups improves the rutting resistance of HEMA [31]. With the increase in the HEUG dosage, the G*/sin*δ* value increases slightly, and the deformation resistance remains at a similar level, which is consistent with the aforementioned analysis.

**Figure 16:**
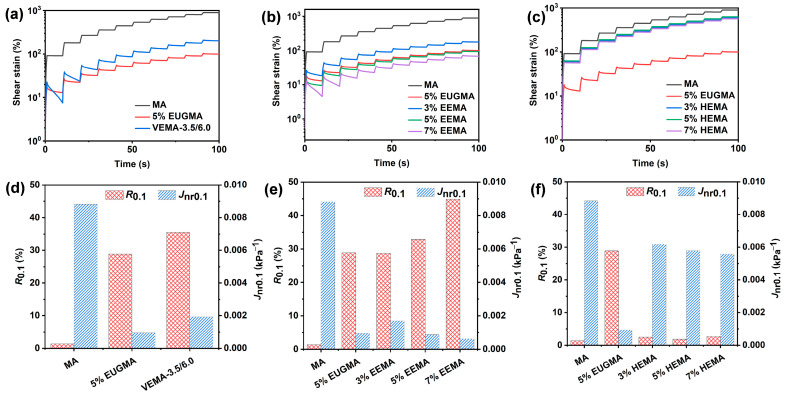
MSCR curves of various asphalts under 0.1 kPa stress level: (**a**) VEMA; (**b**) EEMA; (**c**) HEMA, *J*_nr0.1_ and *R*_0.1_ values: (**d**) VEMA; (**e**) EEMA; (**f**) HEMA.

The multiple stress creep curves, non-recoverable creep compliance (*J*_nr0.1_), and elastic recovery rate (*R*_0.1_) of various asphalts under low stress level (0.1 kPa) are presented in Figure 16. Among these parameters, *J*_nr0.1_ and *R*_0.1_ reflect the permanent deformation resistance and elastic recovery characteristics of asphalt, respectively. The higher the *R* value, the better the elastic recovery; the lower the *J*_nr_ value, the stronger the permanent deformation resistance [42,43]. As can be seen from Figure 16, the shear strain of asphalt increases with the extension of the loading time, and modified asphalts exhibit smaller shear strains than MA, indicating that all modified asphalts have better deformation resistance than MA. The *J*_nr0.1_ of MA is higher than that of modified asphalts, while its *R*_0.1_ is lower, which is because MA mainly behaves as a viscous fluid at high temperatures, with weak intermolecular forces between various components in asphalt. It is prone to deformation under stress and cannot recover to its original state once deformed, thus forming permanent deformation. The addition of modifiers strongly enhances the elasticity of asphalt and inhibits its flow. It can be observed from Figure 16a,d that the cumulative shear strain of VEMA is higher than that of EUGMA, which is because EUG itself has a relatively high modulus, and its incorporation into asphalt endows EUGMA with better deformation resistance. VEMA has the highest *R*_0.1_, and its *J*_nr0.1_ is higher than that of EUGMA but lower than that of MA, demonstrating that after vulcanization, VEMA has better elastic recovery capacity, and its deformation resistance is significantly improved compared with MA. The reason is that the proportion of elastic components in MA increases after vulcanization, and the vulcanized cross-linked network significantly enhances the elasticity of MA, enabling it to recover deformation more quickly and fully after the removal of load. As shown in Figure 16b,e, with the increase in the EEUG dosage, *R*_0.1_ increases continuously, while *J*_nr0.1_ and shear deformation decrease continuously, indicating that the elastic recovery capacity and deformation resistance of EEMA are continuously enhanced with the increase in dosage, showing a trend of becoming a high-elasticity material. This is because, on the one hand, the proportion of elastic components inside EEMA increases with the increase in the EEUG dosage; on the other hand, the number of connection sites between epoxy groups and polar functional groups of asphalt increases with the increase in dosage. The combined effect of these two aspects enhances the elasticity and deformation resistance of EEMA. It can be seen from Figure 16c,f that, with the increase in the HEUG dosage, *R*_0.1_ increases slightly, and *J*_nr0.1_ changes little, indicating that the dosage of HEUG has little effect on the elastic recovery and deformation resistance of HEMA, which is consistent with the results of the temperature sweep test.

**Figure 17:**
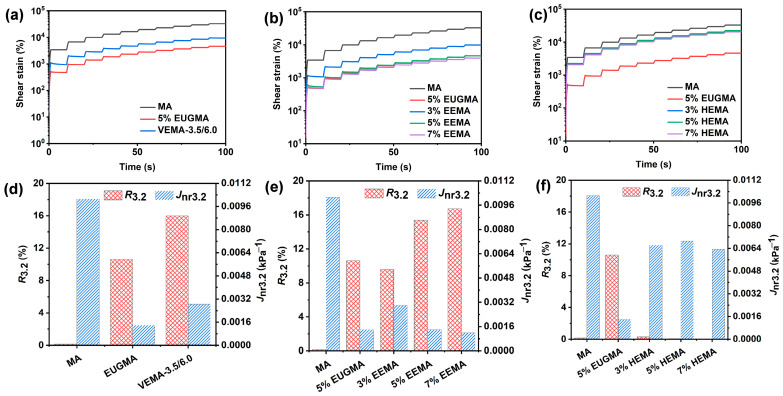
MSCR curves of various asphalts under 3.2 kPa stress level: (**a**) VEMA; (**b**) EEMA; (**c**) HEMA, *J*_nr3.2_ and *R*_3.2_ values: (**d**) VEMA; (**e**) EEMA; (**f**) HEMA.

A high stress level (3.2 kPa) can better simulate the actual service performance of asphalt under heavy traffic loading. The variations in the MSCR curves, *J*_nr3.2,_ and *R*_3.2_ values of various asphalts under high stress levels are presented in Figure 17. It can be seen from the figure that, under high stress, the variation trend of the MSCR curves is consistent with that under low stress levels. Compared with low stress levels, the cumulative shear strain increases, and the *J*_nr3.2_ and *R*_3.2_ values decrease under high stress levels. This indicates that high stress induces a larger deformation of asphalt. Based on the AASHTO M332 standard, the non-recoverable creep compliance *J*_nr3.2_ of all modified asphalts at different dosages is less than 0.5 kPa^−1^. This indicates that the modified asphalts, regardless of the dosage levels, are suitable for “Extremely Heavy” (E) traffic levels, specifically for special road sections subject to extremely high temperatures and long-term high-stress conditions. For VEMA, high stress not only causes large deformation but also damages the internal cross-linked network, resulting in more unrecoverable deformation. For EEMA, high stress exceeds the elastic limit of EEUG, reducing its elastic recovery capacity under high stress. For HEMA, high stress causes irreversible damage to the hydrogen bond network, leading to large deformation and loss of elastic recovery capacity.

#### 3.2.2. Analysis of Low-Temperature Cracking Resistance of Modified Asphalt

**Figure 18:**
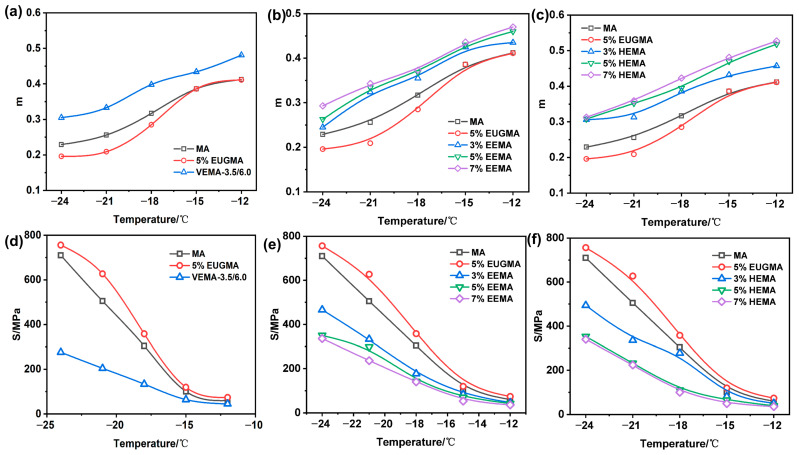
*m* values of various asphalts: (**a**) VEMA; (**b**) EEMA; (**c**) HEMA and *S* values: (**d**) VEMA; (**e**) EEMA; (**f**) HEMA.

The creep stiffness modulus (*S*) and creep rate (*m*) are key parameters to characterize the low-temperature performance of asphalt binder. A smaller *S* value and a larger *m* value correspond to better stress relaxation capacity and low-temperature cracking resistance of asphalt [44]. The *S* and *m* values of all asphalt specimens are plotted in Figure 18. For all tested asphalt samples, the *m* value decreases while the *S* value increases continuously as the test temperature drops. This is because asphalt gradually transforms from a viscoelastic state to a rigid brittle state at low temperatures, leading to a continuous decline in its deformation recovery capacity.

At the same test temperature, EUGMA exhibits the smallest *m* value and the largest *S* value among all samples, indicating the worst low-temperature cracking resistance. This is attributed to the strong crystallization tendency of unmodified EUG molecules at low temperatures. Unlike the flexible elastomer phase at room temperature, these low-temperature crystals significantly increase the stiffness of the asphalt system, thus deteriorating its low-temperature deformation and relaxation properties. As shown in Figure 17d and Figure 18a, at the identical test temperature, VEMA has the highest *m* value and the lowest *S* value among all specimens. This result demonstrates that the low-temperature performance of VEMA is significantly improved compared with both MA and EUGMA. The underlying mechanism is that, in the unvulcanized state, EUG molecules are only uniformly dispersed in the asphalt phase. After the addition of vulcanizing additives, EUG molecular chains are connected into an integral structure via chemical cross-linking, forming a three-dimensional elastic network with excellent elasticity, which effectively enhances the low-temperature stress relaxation and cracking resistance of the binder.

Figure 18b,e show that EEMA has a higher *m* value and lower *S* value than MA at the same test temperature, indicating that the incorporation of epoxidized EUG (EEUG) effectively improves the low-temperature performance of MA. This improvement is attributed to two core factors: on the one hand, the introduction of epoxy groups enhances the interfacial compatibility between EEUG and the base asphalt matrix; on the other hand, epoxy groups inhibit the crystallization of EUG molecular chains, thus enhancing the flexibility of the EEMA system at low temperatures. Furthermore, with the increase in the EEUG dosage, more epoxy groups combine with polar molecules in MA, constructing a tougher, more homogeneous, and more stable internal structure at low temperatures, which further optimizes the low-temperature performance of EEMA. As presented in Figure 18c,f, HEMA exhibits a higher *m* value and lower *S* value than MA at the same test temperature, verifying that the introduction of hydroxylated EUG (HEUG) improves the low-temperature performance of base asphalt. This is because the introduced −OH inhibit the crystallization of EUG molecular chains, avoiding the stiffness increase caused by low-temperature crystallization. Meanwhile, strong hydrogen bonds are formed between adjacent −OH groups, which further connect into a dynamically stable physical cross-linked network. At low temperatures, this hydrogen bond network can dissipate deformation energy through reversible breaking and recombination, effectively preventing crack initiation and propagation caused by stress concentration, thus improving the low-temperature cracking resistance of asphalt. With the increase in the HEUG dosage, the physical cross-linked network formed by −OH groups is further strengthened, leading to continuously enhanced low-temperature performance of HEMA.

**Figure 19:**
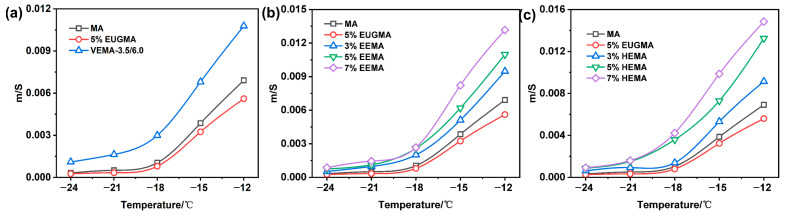
*m*/*S* values of various asphalts: (**a**) VEMA; (**b**) EEMA; (**c**) HEMA.

The ratio of *m*/*S* at 60 s can better evaluate the low-temperature performance of asphalt; the higher the m/S ratio, the stronger the low-temperature cracking resistance of the material [45], as shown in Figure 19. As can be seen from Figure 19, the *m*/*S* ratio of all asphalts decreases with the decrease in temperature. At low temperatures, the mobility of molecular chain segments deteriorates, the asphalt becomes brittle, and its low-temperature cracking resistance decreases. At the same temperature, EUGMA has the worst low-temperature performance, which is because EUG tends to crystallize in low-temperature environments, thus impairing the low-temperature performance of MA. It can be observed from Figure 19a that, at the same temperature, VEMA has the largest *m*/*S* ratio and the best low-temperature cracking resistance. This is because sulfur bridge bonds are introduced after vulcanization, constructing a more stable and elastic network structure, which can better maintain chain segment movement at low temperatures and significantly improve the low-temperature cracking resistance of MA. As shown in Figure 19b, the *m*/*S* ratio of EEMA is higher than that of MA, with favorable low-temperature cracking resistance. This is because the epoxy groups combine with the polar components of MA, maintaining good chain segment mobility and elasticity at low temperatures. With the increase in the EEUG dosage, the number of connection sites with polar functional groups increases, the elasticity is further enhanced, and the low-temperature performance of MA is continuously improved. It can be seen from Figure 19c that the *m*/*S* ratio of HEMA is higher than that of MA, which is because HEUG has good toughness at low temperatures, and its incorporation into MA improves the low-temperature flexibility of the mixture. With the increase in the HEUG dosage, the *m*/*S* ratio increases continuously, and the low-temperature cracking resistance is enhanced, which is because a higher dosage results in better flexibility and thus stronger low-temperature cracking resistance.

The BBR test temperatures of −12 °C and −18 °C are representative of the service conditions in most cold-region road infrastructures. In the Superpave PG system, these test temperatures imply a safety margin, ensuring the pavement resists cracking at actual surface temperatures as low as −28 °C. The significant improvement in the *m*/*S* ratio for VEMA and EEMA compared to MA demonstrates their superior climatic adaptability in high-latitude or high-altitude areas.

#### 3.2.3. Analysis of Microscopic Morphology and Mechanical Properties of Modified Asphalt

**Figure 20:**
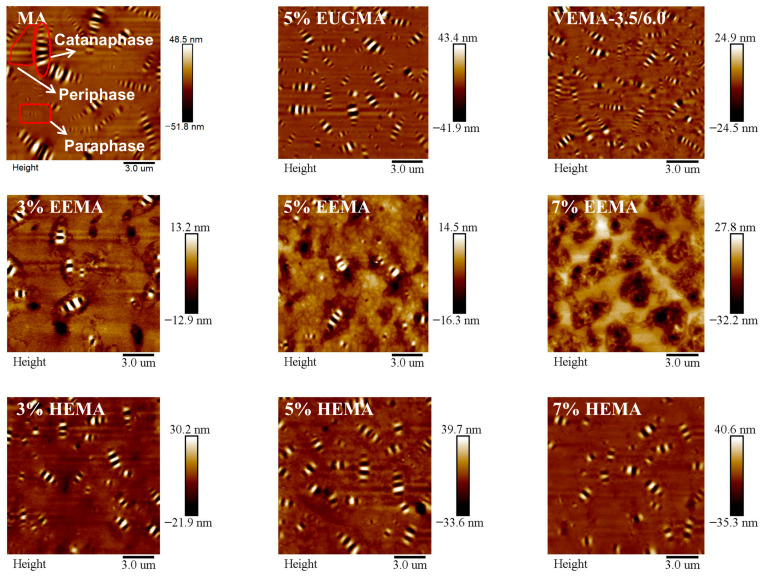
AFM morphology images of various asphalts.

As can be seen from Figure 20, the surface of MA exhibits distinct bee-like phase, continuous phase, and intermediate phase. Among them, the bee-like phase shows a bright–dark alternating phenomenon, which is the result of the interaction between crystallized wax and other components in asphalt. Asphaltenes act as the core of the bee-like structure, while wax serves as the inducer of the bee-like structure; the relative contents of these two components exert a significant influence on the distribution and size of the bee-like structure. The continuous phase closely surrounds the bee-like phase, forming a continuous edge region between the bee-like phase and the dispersed phase. Dominated by aromatics and polar aromatics and possibly containing some resins, it acts as a transition zone between asphaltenes and the continuous matrix. The dispersed phase is the continuous matrix of asphalt, filling the space between the bee-like phase and the continuous phase. Mainly composed of saturates and low-polarity aromatics, it is the most fluid part of asphalt [46]. After adding EUG and VEUG into MA, the size of the bee-like structures decreases, while their quantity increases. Compared with EUGMA, VEMA has a larger number of bee-like structures with a smaller size and denser distribution, which may be because a cross-linked network is formed inside VEMA after vulcanization, restricting the aggregation and coalescence rate of asphaltenes and making it difficult to form large aggregates. It can also be found that the number of bee-like structures in EEMA decreases significantly, the integrity of the bright–dark alternating bee-like structures is damaged, leaving almost only the bright parts, and this phenomenon becomes more pronounced with the increase in the EEUG dosage. Further analysis shows that with the increase in the HEUG dosage, the quantity and size of the bee-like structures also increase slightly. This is because −OH groups in HEUG are strong polar functional groups, and their amount increases with the rise in dosage, which significantly enhances intermolecular interactions and aggregation behavior. The originally disorderly dispersed tiny HEUG particles will aggregate into larger structures through polar attraction.

**Figure 21:**
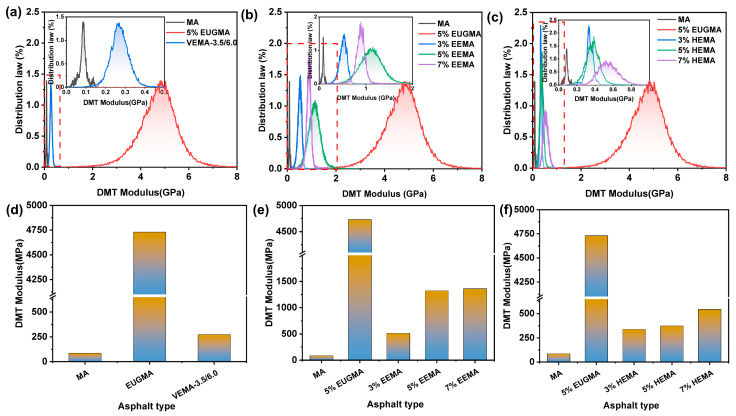
Statistical histograms of DMT modulus of various asphalts: (**a**) VEMA; (**b**) EEMA; (**c**) HEMA, and DMT modulus values: (**d**) VEMA; (**e**) EEMA; (**f**) HEMA.

The DMT modulus reflects the deformation resistance of asphalt [47]. The distribution and magnitude of the DMT modulus of various asphalts are shown in Figure 21. As can be seen from Figure 21, the DMT modulus of all asphalts follows a normal distribution. EUGMA exhibits the highest DMT modulus, indicating that EUG itself has a relatively high modulus, which also endows EUGMA with a high modulus when incorporated into asphalt. This is consistent with the results of the temperature sweep and MSCR tests. It can be observed from Figure 21a,d that the DMT modulus distribution band of VEMA is wider than that of MA. This is because the cross-linked network acts as a skeleton in asphalt, thereby increasing the stiffness of asphalt and enhancing its deformation resistance. In addition, the DMT modulus of VEMA is higher than that of MA, demonstrating that it has the strongest deformation resistance. Furthermore, the DMT modulus of VEMA is reduced by 94% compared to EUGMA. As shown in Figure 21b,e, with the increase in the EEUG dosage, the DMT modulus distribution band continuously shifts to the right, and the DMT modulus increases by 502.3%, 1454.1%, and 1502.3%, respectively, compared with MA. This is because, on the one hand, the proportion of elastic components in EEMA increases with the rise in EEUG dosage, leading to an increase in the DMT modulus; on the other hand, more epoxy groups form covalent bonds with the polar groups in MA as the EEUG dosage increases, which further raises the DMT modulus of asphalt. The combined effect of these two aspects results in an increase in DMT modulus with the increase in EEUG dosage. As can be seen from Figure 21c,f, compared with MA, the DMT modulus of HEMA increases by 296.4%, 340.2%, and 536.5%, respectively, with the increase in the HEUG dosage, but the growth rate is relatively low. This is mainly because HEUG is a soft elastomer; so, the increment of the DMT modulus is limited.

**Figure 22:**
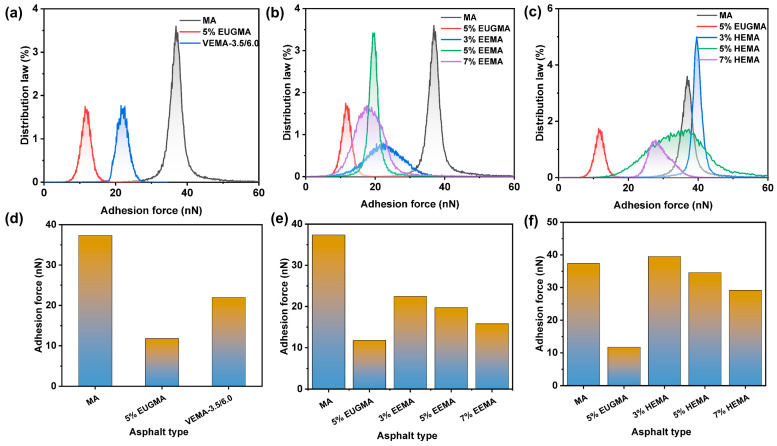
Statistical histograms of adhesion force of various asphalts: (**a**) VEMA; (**b**) EEMA; (**c**) HEMA and adhesion force values: (**d**) VEMA; (**e**) EEMA; (**f**) HEMA.

The adhesion force on the asphalt surface measured by AFM refers to the interaction force between the molecules on the asphalt surface and the molecules at the tip of the probe, mainly including electrostatic force, van der Waals force, and capillary force. It reflects the adhesion between the probe tip and asphalt and is an important parameter in the microscopic mechanical testing of asphalt [48]. As can be seen from Figure 22a,d, the adhesion force distribution curve of VEMA follows a normal distribution with a unique peak, indicating that the modifier is relatively uniformly distributed, and the material has a stable phase structure. The adhesion of VEMA is 87% higher than that of MA, indicating that the addition of EUG and vulcanizing agent can improve the adhesion capacity of asphalt. This is because VEMA forms a three-dimensional cross-linked network, which inhibits the phase separation between EUG and asphalt and results in a more uniform microstructure. As can be seen from Figure 22b,e, compared with MA, the adhesion of EEMA decreases by 40.5%, 48.6%, and 59.4%, respectively, with the increase in the EEUG dosage. This is because the epoxy groups of EEMA form epoxy bonds with asphalt components, leading to large energy dissipation during the probe contact–separation process, which is manifested as a high adhesion force. However, the higher the EEUG dosage, the larger the DMT modulus, the harder the asphalt, and the continuously decreasing adhesion force. As can be seen from Figure 22c,f, the adhesion force of HEMA decreases with the increase in the HEUG dosage, but the reduction range is smaller than that of EEMA. This is because HEUG is a soft elastomer; although the increase in its dosage will lead to an increase in DMT modulus, the increment is small, thus maintaining good adhesion force.

## 4. Conclusions

Three types of chemically modified EUG were prepared, their properties were characterized, and they were applied to asphalt materials for road engineering, with the modification effects of the asphalts evaluated. On this basis, the following conclusions can be drawn:

(1) Increasing the sulfur dosage promotes C=C bond participation in vulcanization, forming C-S cross-links and a sulfur-bridged network in VEUG. For EEUG, the epoxidation degree *E* (mol%) rises with the H_2_O_2_ dosage; peroxy acid attack on C=C bonds causes EUG chain scission, *M*_n_ and *M*_w_, and concurrent epoxy group formation. Glacial acetic acid induces epoxy ring-opening and hydroxyl grafting to form HEUG, whose hydroxylation degree *H* (mol%) increases with the reaction time; higher *H* (mol%) continuously reduces *M*_n_ and *M*_w_ of HEUG, with a narrower molecular weight distribution.

(2) A higher vulcanization degree enhances the cross-linked network’s suppression of EUG chain regularity, reducing the crystallinity and increasing *T*_g_, while thermal degradation of the cross-linked network lowers VEUG’s initial thermal stability. For EEUG and HEUG, increasing modification degree disrupts EUG chain regularity and symmetry, leading to monotonic *T*_g_ elevation, reduced crystallinity, improved thermal stability, and enhanced hydrophilicity. Notably, EEUG with *E* > 18.23 mol% and all HEUG samples show a complete loss of crystallinity.

(3) Increasing the vulcanization degree synchronously enhances the tensile strength and elongation at break of VEUG, driving a rigid–plastic to elastic transition. For EEUG, higher *E* (mol%) reduces the elastic modulus, tensile strength, and tear strength, while improving the ductility and promoting the same rigid–plastic to elastic transition. HEUG exhibits complete suppression of plastic deformation via dynamic hydrogen bond breakage and recombination, featuring soft elasticity, large deformability, a gently rising stress-strain response, and significantly reduced hardness.

(4) Compared with MA, the cross-linked network in VEMA yields significantly higher G*/sin*δ* and *R*, lower *J_nr_* at high temperatures, and an elevated *m*/*S* ratio at low temperatures, indicating simultaneously enhanced high-temperature deformation resistance and low-temperature cracking resistance. For EEMA, increasing modifier loading continuously increases G*/sin*δ*, *R*, and *m*/*S* while reducing *J_nr_*, delivering progressively improved high- and low-temperature performance. HEMA also improves asphalt’s thermal and mechanical performance, with increasing HEUG loading yielding moderate high-temperature improvement but pronounced enhancement in low-temperature cracking resistance.

(5) The cross-linked network in VEMA inhibits wax self-aggregation, generating more abundant finer bee-like microstructures, along with an increased DMT modulus and adhesion. EEUG disrupts bee-like structures via interactions between epoxy groups and polar asphaltene functional groups; higher modifier loading increases the modulus and reduces adhesion. HEUG restricts asphaltene aggregation through a hydroxyl-mediated intermolecular cross-linked network, causing a slight increase in the number and size of bee-like structures. While the DMT modulus increases with HEUG loading and the adhesion decreases accordingly, high HEUG loadings still maintain favorable adhesion.

The research plan for the next stage is as follows. As a natural bio-based material, the eco-friendly property of EUG is one of its core competitive advantages. Future work will be dedicated to scaling up the lab-scale chemical modification process to industrial mass production and evaluating the yield stability and economic feasibility of the modifier in large-scale production. Meanwhile, it is recommended to conduct a full life cycle assessment and cost assessment, to quantitatively analyze the comprehensive benefits of asphalt pavement modified with chemically modified EUG in terms of carbon footprint reduction, resource recycling, and preventive maintenance cost reduction, to provide a decision-making basis for its large-scale application in the construction of green transportation infrastructure.

## Figures and Tables

**Table 1:** Technical specifications of experimental chemicals.

Name	Purity (%)	*M*_F_	*M*_W_	Density (g/cm^3^)	Boiling Point (°C)	Melting Point (°C)	Flash Point (°C)
Sulfur	99.98	S	32.07	2.07	445	114	168
SA	≥99.5	C_18_H_36_O_2_	284.48	0.84	361	69.6	196
ZnO	99.99	ZnO	81.39	5.68	1949	1975	27
CBS	≥98	C_13_H_16_N_2_S_2_	264.41	1.31	410	100	202
IPPD	≥95	C_15_H_18_N_2_	226.32	1.1	180	85	172
HP	30	H_2_O_2_	34.01	1.11	106	−0.42	/
FA	98	CH_2_O_2_	46.03	1.22	100	8.2	69
Xylene	99	C_8_H_10_	106.17	0.86	144	−25.2	25
GAA	99	C_2_H_4_O_2_	60.05	1.1	117	16.6	39
Ethanol	95	CH_3_CH_2_OH	46.07	0.79	78	−114	12

Notes: SA: stearic acid; ZnO: zinc oxide; CBS: N-cyclohexyl-2-benzothiazole sulfonamide; IPPD: N-isopropyl-N’-Phenyl-p-phenylenediamine; HP: hydrogen peroxide; FA: formic acid; GAA: glacial acetic acid; *M*_F_: molecular formula; *M*_W_: molecular weight.

**Table 2:** Technical specifications of MA.

Test Items	Unit	Result	Requirements	Testing Method
Penetration (25 °C, 100 g, 5 s)	0.1 mm	85	80~100	JTG E20 T 0604
Penetration Index	/	−1.3	−1.5~+1.0	JTG E20 T 0604
Softening point (Global method)	°C	45	≥44	JTG E20 T 0606
Dynamic viscosity (60 °C)	Pa·s	151	≥140	JTG E20 T 0620
Ductility (10 °C, 5 cm/min)	cm	>100	≥30	JTG E20 T 0605
Ductility (15 °C, 5 cm/min)	cm	>150	≥100	JTG E20 T 0605

**Table 3:** Vulcanization formulation.

VEUG Type	EUG/Parts	SA/phr	ZnO/phr	CBS/phr	IPPD/phr	Sulfur/phr
VEUG-1.6	100	2	5	2	2	1.6
VEUG-1.8	100	2	5	2	2	1.8
VEUG-2.0	100	2	5	2	2	2.0
VEUG-2.2	100	2	5	2	2	2.2
VEUG-2.4	100	2	5	2	2	2.4

**Table 4:** Effects of reaction conditions on *H* (mol%).

Temperature (°C)	Time (h)	pH	*H* (mol%)
110	1.0	3	13.30
110	2.5	3	15.77
110	4.0	3	17.79
110	5.5	3	20.75
110	7.0	3	21.05
110	5.0	3	19.70
110	5.0	4	14.60
120	5.0	3	13.15
130	5.0	3	19.92

**Table 5:** Molecular weight and polydispersity index of chemically modified EUG.

Type	*M*_n_ (g/mol)	*M*_w_ (g/mol)	PDI
EUG	281,638	657,991	2.336
EEUG-8.96%	272,109	627,531	2.036
EEUG-12.22%	263,956	592,162	2.243
EEUG-18.23%	242,620	542,992	2.238
EEUG-22.98%	190,498	355,186	1.865
EEUG-26.67%	173,513	336,820	1.941
HEUG-13.30%	71,895	120,554	1.677
HEUG-15.77%	67,069	105,231	1.569
HEUG-17.79%	25,502	38,235	1.499
HEUG-20.75%	19,545	25,607	1.310
HEUG-21.05%	16,422	19,356	1.179

**Table 6:** Crystallization-melting temperatures of modified EUG.

Type	*T*_m_ (°C)	*T*_g_ (°C)	*T*_c_ (°C)	Δ*H* (J/g)	*X*_c_* (%)
EUG	49.14	−70.38	18.19	39.95	31.74
VEUG-1.6	43.00	−60.64	14.52	35.18	27.95
VEUG-1.8	42.05	−60.16	12.49	34.37	27.30
VEUG-2.0	41.84	−59.04	12.41	31.61	25.11
VEUG-2.2	41.58	−58.67	11.06	31.22	24.80
VEUG-2.4	38.63	−58.35	10.66	30.85	24.50
EEUG-8.96%	41.12	−59.16	17.25	29.45	23.39
EEUG-12.22%	32.40	−57.1	4.56	29.14	23.15
EEUG-18.23%	/	−54.64	/	1.91	1.52
EEUG-22.98%	/	−48.94	/	0.20	0.16
EEUG-26.67%	/	−41.48	/	/	/
HEUG-13.30%	/	−43.89	/	/	/
HEUG-15.77%	/	−40.83	/	/	/
HEUG-17.79%	/	−35.06	/	/	/
HEUG-20.75%	/	1.54	/	/	/
HEUG-21.05%	/	7.72	/	/	/

**Table 7:** Thermal decomposition parameters of various modified EUG samples.

Type	*T*_onset_/°C	*T*_5%_/°C	*T*_10%_/°C	*T*_max_/°C	CY/%
EUG	314.86	316.36	342.50	377.29	2.95
VEUG-1.6	302.61	311.35	335.90	374.40	3.59
VEUG-1.8	298.31	306.23	332.00	372.40	3.53
VEUG-2.0	296.53	305.95	333.11	374.31	5.3
VEUG-2.2	288.00	290.61	336.49	375.43	5.61
VEUG-2.4	279.32	289.82	339.40	376.90	7.07
EEUG-8.96%	315.63	324.97	346.91	377.61	2.73
EEUG-12.22%	318.55	321.18	349.21	383.13	2.12
EEUG-18.23%	323.68	329.89	355.50	386.85	2.44
EEUG-22.98%	322.55	329.87	357.37	390.22	2.38
EEUG-26.67%	335.48	340.51	362.85	389.84	2.17
HEUG-13.30%	228.94	247.67	340.30	372.31	2.77
HEUG-15.77%	242.25	256.58	287.84	386.04	1.65
HEUG-17.79%	243.25	259.80	306.33	380.71	3.15
HEUG-20.75%	259.26	271.67	288.37	403.85	0.92
HEUG-21.05%	282.25	290.23	334.81	377.21	1.66

**Table 8:** Mechanical properties of modified EUG.

Type	*T*_s_ (N/mm)	*σ* (MPa)	ε (%)	σ_100%_ (MPa)	*σ*_200%_	*σ*_300%_	Shore A	*a*_i_ (kJ/m^2^)	*σ*_c_ (MPa)
EUG	85.29	24.51	270.6	10.97	18.89	0	84.30	15.82	20.45
VEUG-1.6	95.42	21.13	281.0	10.49	16.18	/	82.10	15.87	17.72
VEUG-1.8	82.94	20.92	296.3	8.75	14.89	/	81.20	16.98	17.06
VEUG-2.0	81.64	19.07	298.6	8.27	13.60	/	80.20	17.30	16.45
VEUG-2.2	81.96	17.35	305.5	6.71	11.77	17.13	78.20	21.99	15.96
VEUG-2.4	82.35	16.48	306.1	6.31	11.09	16.31	75.30	22.36	15.74
EEUG-8.96%	77.77	10.18	143.6	8.37	/	/	66.20	12.47	12.61
EEUG-12.22%	74.83	10.02	182.7	6.50	/	/	63.20	11.01	10.85
EEUG-18.23%	76.89	8.45	221.9	4.66	7.82	/	60.40	10.80	10.53
EEUG-22.98%	75.51	7.69	240.3	6.57	7.05	/	56.70	10.65	7.11
EEUG-26.67%	78.21	6.36	262.0	3.03	4.90	/	55.40	8.40	4.77
HEUG-13.30%	13.04	1.99	1360.3	0.83	0.89	0.96	49.20	/	1.52
HEUG-15.77%	9.81	1.90	941.7	0.83	0.90	1.02	48.30	/	1.23
HEUG-17.79%	10.69	1.77	799.3	0.91	1.10	1.23	46.70	/	1.18
HEUG-20.75%	11.28	1.59	570.5	0.92	1.17	1.34	45.50	/	1.10
HEUG-21.05%	7.94	0.65	679.9	0.58	0.60	0.61	40.30	/	1.08

## Data Availability

The original contributions presented in this study are included in the article. Further inquiries can be directed to the corresponding authors.
